# A comprehensive summary of the impact of the COVID era on various gastrointestinal cancers

**DOI:** 10.1002/ags3.12811

**Published:** 2024-04-25

**Authors:** Koshi Mimori

**Affiliations:** ^1^ Kyushu University Beppu Hospital Beppu Japan

The COVID era has ended as the pandemic subsided. It is anticipated that cancer care was hindered during the COVID era, which we expect had various negative effects. But what was the situation in actual clinical practice? Various reports have been published. In this issue, two reports have been made on the relationship between the treatment of gastrointestinal diseases and the pandemic. One is the impact of COVID‐19 infection on colorectal perforation by Ogawa et al. They reported that among 13 107 cases of colorectal perforation, the 30‐day postoperative mortality was 1371 cases (10.5%), and there was no difference compared to the pre‐COVID era (DOI: 10.1002/ags3.12758). Furthermore, Akagi et al. clarified that the standard mortality rates for laparoscopic distal gastrectomy (LDG) for gastric cancer and laparoscopic low anterior resection (LLAR) for rectal cancer did not differ from the pre‐COVID era, indicating that laparoscopic surgery was safely performed in Japan (DOI: 10.1002/ags3.12776). This editorial summarizes the impacts of the COVID era and pandemic on various gastrointestinal cancers by type of cancer.

Colorectal Cancer: 48 900 CRC patients were extracted from the Dutch cancer registry. Compared to the same period before COVID, CRC patients decreased by up to 36%. In particular, during the first peak (weeks 12–20 of 2020), Stage I decreased by 4%, while Stage IV increased by 7%.^1^ In Japan, it has also been reported that the number of CRC screening participants has not returned to the pre‐pandemic levels, with decreases of −13.4% (2020) and −7.3% (2021).^2^

Gastric Cancer: A single institution in Korea compared a pre‐pandemic surgical group (99 cases) with a during‐pandemic group (118 cases) and found that while short‐term outcomes and long‐term complications were equivalent, perioperative outcomes were poorer during the pandemic.^3^ Takeuchi and colleagues observed a decrease in 568 cases of gastrectomy. However, they reported that mortality and perioperative outcomes during the pandemic did not worsen compared to before the pandemic.^4^

Esophageal Cancer: In the UK, diagnoses, management, and outcomes of esophageal cancer were investigated before and after the COVID‐19 lockdown. Overall survival was 9.9 months before the lockdown and 6.9 months after, significantly worse post‐lockdown.^5^ It is speculated that this was due to delayed examinations during the lockdown, leading to esophageal cancer being detected at more advanced stages. However, reports from Japan indicated similar (not inferior) outcomes to pre‐COVID times, despite limited medical resources.^6^

Liver Cancer: Munoz‐Martinez and colleagues reported a 2.2% decrease in mortality rates for HCV (and HBV)‐related HCC, while mortality rates for NAFLD (alcohol‐related liver disease)‐related hepatocellular carcinoma increased by 3%. Delays in diagnosis were reported at 80.9%. Additionally, the 30‐day mortality was 15% for SARS‐CoV‐2‐related deaths, compared to 3.7% for non‐SARS‐CoV‐20related deaths.^7^

Pancreatic Cancer: During the lockdown, referral rates decreased by 29%. However, there were no differences in metastasis rates (*p* = 0.39), TNM (*p* = 0.80), or treatment choices (*p* = 0.94) between pre‐pandemic and during‐pandemic periods. Moreover, there was no significant difference in the 1‐year survival rate between pre‐pandemic (2019) and during‐pandemic (2020–21) periods for surgery, chemotherapy, or best supportive care (BSC).^8^

In summary, the consultation and referral rates for patients with gastrointestinal cancer decreased due to the pandemic, and early diagnosis was delayed. For all cancer types, those global delays in screening, diagnosis, and treatment will potentially lead to an increase in cancer‐related deaths in the future, therefore, we need to have a necessitating careful long‐term observation. Most of the reports indicated that the perioperative management of gastrointestinal cancers in Japan was excellent generally, with no difference in safety before and after the pandemic.

## CONFLICT OF INTEREST STATEMENT

The author declare no conflicts of interest for this article.
